# Significant Associations of IgG Glycan Structures With Chronic Graft-Versus-Host Disease Manifestations: Results of the Cross-Sectional NIH Cohort Study

**DOI:** 10.3389/fimmu.2021.633214

**Published:** 2021-07-14

**Authors:** Ema Prenc, Drazen Pulanic, Maja Pucic-Bakovic, Ivo Ugrina, Lana Desnica, Milan Milosevic, Filip Pirsl, Sandra Mitchell, Jeremy Rose, Radovan Vrhovac, Damir Nemet, Gordan Lauc, Steven Z. Pavletic

**Affiliations:** ^1^ Fidelta Ltd., Translational Research and Alliances, Zagreb, Croatia; ^2^ School of Medicine, University of Zagreb, Zagreb, Croatia; ^3^ Department of Internal Medicine, Division of Hematology, University Hospital Centre Zagreb, Zagreb, Croatia; ^4^ Genos Ltd., Zagreb, Croatia; ^5^ Faculty of Science, University of Split, Split, Croatia; ^6^ Department of Environmental and Occupational Health and Sports, Andrija Stampar Teaching Institute of Public Health, Zagreb, Croatia; ^7^ Center for Cancer Research, National Cancer Institute, National Institutes of Health, Bethesda, MD, United States; ^8^ Division of Cancer Control and Population Sciences, National Cancer Institute, National Institutes of Health, Bethesda, MD, United States

**Keywords:** allogeneic hematopoietic stem cell transplantation, chronic graft-versus-host disease, immunoglobulin G, glycans, biomarker

## Abstract

Chronic graft-versus-host disease (cGvHD) is a systemic alloimmune and autoimmune disorder and a major late complication of allogeneic hematopoietic stem cell transplantation (alloHSCT). The disease is characterized by an altered homeostasis of the humoral immune response. Immunoglobulin G (IgG) glycoprotein is the main effector molecule of the humoral immune response. Changes in IgG glycosylation are associated with a number of autoimmune diseases. IgG glycosylation analysis was done by the means of liquid chromatography in the National Institutes of Health (NIH) cohort of 213 cGvHD patients. The results showed statistically significant differences with regards to cGvHD NIH joint/fascia and skin score, disease activity and intensity of systemic immunosuppression. ROC analysis confirmed that IgG glycosylation increases specificity and sensitivity of models using laboratory parameters and markers of inflammation associated with cGvHD (eosinophil count, complement components C3 and C4 and inflammation markers: albumin, CRP and thrombocyte count). This research shows that IgG glycosylation may play a significant role in cGvHD pathology. Further research could contribute to the understanding of the disease biology and lead to the clinical biomarker development to allow personalized approaches to chronic GvHD therapy.

## Introduction

Chronic graft-versus-host disease (cGvHD) is a serious late complication of the allogeneic hematopoietic stem cell transplantation (alloHSCT) associated with an increased non-relapse mortality rate, disturbed physical and functional status and reduced life quality of surviving patients. The disease affects approximately 50% of patients after alloHSCT with clinical features resembling aspects of many different autoimmune disorders. It is caused by a disparate immunological system interacting with a new (and perceivably foreign) host environment in a potentially curative attempt to replace the diseased immunity with a graft from a healthy donor and produce the protective graft-versus-tumor effect. The whole mechanism of this multisystemic disease remains to be elucidated (1). The disease is characterized, among other, by an altered homeostasis of the humoral immune response and the production of allo-/auto-antibodies.

The National Institutes of Health (NIH) consensus on cGvHD in 2005 (and updated in 2014) enabled standardized and systematic approach to cGvHD and uniform collection of clinical data (2, 3), and also defined the basic principles for biomarker development (4, 5). Despite some promising plasma biomarkers and cellular subpopulations being identified which should be further studied in verification studies (4), to date research has not provided the necessary biomarker whose activity matches clinical observations of severity and/or disease activity. This remains one of the major obstacles in cGvHD research: the absence of suitable biomarkers that would predict disease occurrence, or provide more accurate initial diagnosis, as well as prognose or assess its therapeutic response. In general, the search for a suitable cGvHD biomarker is greatly delayed compared to that of the acute GvHD due to the wider range of manifestations, large variation among patients in terms of time to onset and severity, overlap with acute form, and the insufficient number of patients available for clinical studies and verification.

Modern experimental methods and recent exponential development of the so called *–omics* technologies enable researchers to analyze a large number of samples in a short time period, characteristics most desirable when discussing tracking of the disease dynamics. In the course of the quest for a cGvHD biomarker, the advancement of modern -*omics* tools should be exploited. One such tool is glycomics, which has been declared a research priority over the next decade since it has been recognized that glycans are directly involved in the pathophysiology of any major disease and that the further development of this scientific discipline is necessary to achieve the goals of personalized medicine (6).

Glycans are non-linear branched oligosaccharides directly involved in almost every biological process. Glycoproteins are glycoconjugates in which glycans are covalently linked to a polypeptide backbone, leading to the most structurally diverse posttranslational modification of proteins, affecting its conformation and its biological functions (7). One of the most analyzed glycoprotein is immunoglobulin G (IgG), the most abundant class of antibody in the human plasma and the main effector molecule of the humoral immune system (8). The IgG glycan consists of the biantennary heptameric core (three mannose and four N-acetylglucosamine residues) and possible additions of N-acetylglucosamine, fucose, galactose, and sialic acid residues. Effector functions of IgG can be completely changed by the addition or removal of a single monosaccharide residue from its glycans, thus affecting its ability to bind to Fc receptors of various immune cells (9). For example, lack of core fucose increases affinity for FcγRIIIa receptor leading to an improved effector function (10).

Changes in IgG glycosylation are associated with a number of inflammatory conditions, autoimmune and hematological diseases (11, 12) with promising results in the biomarker research and disease pathophysiology understanding, thus suggesting its huge research potential also in the field of GvHD (13).

In this work we analyzed an association of IgG glycan structures with clinical manifestations of a well annotated large cohort of cGvHD patients.

## Materials and Methods

Blood plasma samples of cGvHD patients were collected from 2004-2014, as a part of a clinical cross-sectional study “*Natural History Study of Clinical and Biological Factors Determining Outcomes in Chronic Graft-Versus-Host Disease*” (04-C-0281, clinicaltrials.gov identifier: NCT00331968) conducted at Center for Cancer Research, National Cancer Institute, NIH). Before entering the study, all subjects signed an informed consent approved by the NIH Ethical Committee. According to the study protocol, all subjects (aged 1-75) went through the 4-day multidisciplinary evaluation of cGvHD. For each patient a detailed medical history was collected, including demographics (gender, age), pre- and post-transplant details (primary disease and its status at the moment of transplantation and cGvHD evaluation, pre-transplant conditioning, early complications, previous acute GvHD, infections), donor and graft information (donor’s gender and age, HLA matching, stem cell source) and cGvHD characteristics (disease onset, classification, symptoms, previous and current therapy). Subjects who had received intravenous immunoglobulins (IVIg) within 3 months prior to study entry were excluded from this study, to avoid interference with patients’ IgG glycan analysis. After a physical examination by hematologist, a series of specialist examinations were done along with extensive laboratory processing that included the determination of laboratory markers of inflammation. Cryopreserved samples of heparinized blood plasma were sent to Genos Ltd. (Zagreb, Croatia). IgG was isolated, deglycosylated and glycans analyzed by hydrophilic-interaction ultra-high performance liquid chromatography (HILIC-UHPLC). These methods were described in detail in previous publications (14, 15). The results of UHPLC analysis were in a form of 24 chromatograpic peaks (directly measured parameters). For every directly measured parameter (glycan peak, GP) a percentage of the total IgG glycome was calculated. Additionally, derived parameters (IGP) were determined which represent the share of a group of glycans of similar structural characteristics in the total IgG glycome (e.g. IGP25: the percentage of sialylation of fucosylated galactosylated structures with bisecting N-acetylglucosamine (GlcNAc) in total IgG glycans). Differences in abundances of IgG glycan traits were also observed: fucosylated glycans (all structures with core fucose), galactosylated and agalactosylated glycans [all structures with/without antennary galactose(s)], sialylated glycans (all structures with one or two terminal antennary sialic acids) and glycans with bisecting GlcNAc attached to the glycan core.

Disease NIH cGvHD scores, both global (mild, moderate and severe) and for individual organs (skin, mouth, eyes, liver, gastrointestinal tract, liver, joints/fascia, genital tract for women; none, mild, moderate and severe) were determined according to the established 2005 NIH classification for cGvHD (2). Disease activity was defined by previously described scales (16): clinician’s impression of activity (active versus non-active disease), intensity of systemic immunosuppression at the time of evaluation (1-4) and therapeutic intent at the time of evaluation. Disease was considered more active if the need for systemic immunosuppression was higher or if the practitioner decided to increase systemic therapy due to worsening disease, to substitute systemic therapy due to lack of response or withdraw systemic therapy due to lack of response.

### Statistical Methods

The experimental design was carried out using appropriate methods (such as randomization) where gender and age parameters were taken into account. The results of the experiment went through quality checks using replicates and standards before statistical analysis. Appropriate descriptive statistics (median, range, maximum and minimum values) was used in the statistical processing of the obtained results.

Since IgG glycans often deviate from the normal (Gaussian) distribution, univariate analysis was performed using the non-parametric tests (Wilcoxon) with a goal to detect significant glycan structures. The significance level for the p value was adjusted according to the Bonferroni correction for multiple testing. The results were considered significant if the p value was less than 0.05.

Because glycans are largely correlated, principal components analysis (PCA) method was used to obtain condensed uncorrelated glycan variables (glycans’ principal components, ‘*glycansPC*’). This method enables transforming a large set of variables into a smaller one that still contains most of the information in the large set. A large number of collected laboratory parameters (**Supplementary Table S1**) was reduced as follows: individual clinical laboratory parameters of interest related to cGvHD identified by literature search [eosinophil count, C3, C4 and inflammatory markers: albumin, CRP and platelet count (16)] while the rest of laboratory parameters were reduced to an uncorrelated set of variables by the means of PCA (‘*labsPC*’ variables). These variables were used for further analysis by logistic regression in order to assess the discriminatory potential of IgG glycans in the detection of presence/absence of cGvHD in individual organ systems, and disease activity (active/non-active). Scores describing disease severity and activity were also reduced to fewer categories as follows (also see **Table 1**):

-cGvHD disease score:
 ○ Individual organ NIH cGvHD scores: patients with mild, moderate and severe specific organ involvement were grouped together and compared to patients with no corresponding organ involvement
 ○ Global NIH cGvHD score: patients with mild and moderate cGvHD were grouped together and compared to patients with severe form
-cGvHD activity score:
 ○ intensity of systemic immunosuppression at the time of evaluation: patients with no immunosupression were grouped with the ones categorized as mild intensity, and compared to patients with moderate and high intensity
 ○ therapeutic intent at the time of evaluation: patients whose therapy was decreased or remained unchanged were compared to patients whose therapy was needed to be replaced or discontinued due to inadequate response
 ○ clinician’s impression of activity: patients with active versus patients with non-active disease (irrespective of the topical or systemic immunosupression).

**Table 1:** Characteristics of cGvHD patients and their clinical laboratory parameters.

Characteristics of cGvHD patients				
Gender	Female, *N (%)*	92 (43.2%)		
	Male, *N (%)*	121 (56.8%)		
Age (years), *median (range)*		45 (5-71)		
Time from transplantation until study participation (days), *median (range)*		1136 (131-7746)		
Time from transplantation until cGvHD diagnosis (days), *median (range)*		233 (41-4325)		
Time from cGvHD diagnosis until study participation (days), *median (range)*		756 (0-6545)		
**Pre- and post-transplant information**		**N**	**%**	
Primary disease	Leukemia^a^	138	64.8%	
	Lymphoproliferative disease^b^	60	28.2%	
	Other^c^	15	7.0%	
Myeloablative conditioning	Yes	121	56.8%	
	No	89	41.8%	
	Unknown	3	1.4%	
Donor relation	Related	128	60.1%	
	Unrelated	85	39.9%	
Hematopoietic stem cells source	Bone marrow	41	19.3%	
	Peripheral blood	166	77.9%	
	Umbilical blood	6	2.8%	
Previous acute GvHD	Yes	141	66.2%	
	No	71	33.3%	
	Unknown	1	0.5%	
**Characterization of cGvHD**		**N**	**%**	
cGvHD onset	*De novo*	71	33.3%	
	Progressive	78	36.6%	
	Quiescent	63	29.6%	
	Unknown	1	0.5%	
cGvHD classification	Classic	183	85.9%	
	Overlap	27	12.7%	
	Unknown	3	1.4%	
Lines of systemic therapy, *median (range)*	4 (0-9)			
Clinician’s impression of activity^d^	Active	101	47.4%	
	Age (years), *median (range)*		46.5 (5-71)	
	Non-active	19	8.9%	
	Age (years), *median (range)*		25 (7-66)	
	Unknown	93	43.7%	
Intensity of imunosupression	None/Mild	64	30.0%	
	Moderate/High	149	70.0%	
Activity by therapeutic intent at the time of evaluation	Non-active	72	33.8%	
	Active	93	43.7%	
	Unknown/Not applicable^e^	48	22.5%	
Organs involved with cGvHD	Skin	163	76.5%	
	Mouth	141	66.2%	
	Eyes	166	77.9%	
	Liver	111	52.1%	
	Gastrointestinal tract	92	43.2%	
	Lungs	164	77.0%	
	Joints/fascia	130	61.0%	
	Genital tract (for women)	53	57.6%	
Global cGvHD NIH score	Mild	4	1.9%	
	Moderate	52	24.4%	
	Severe	157	73.7%	
Number of involved organs with cGvHD, *median (range)*	5 (1-8)			
**Laboratory parameters**				
Parameter	*Median (range)*	Parameter	*Median (range)*	
Erythrocyte sedimentation rate (mm/h)	16 (1-113)	Beta 2 microglobulin (mg/L)	2.10 (0.9-70.74)	
Eosinophils (x10^9 /L)	0.08 (0-91.07)	C3 (g/L)	1.35 (0.64-2.22)	
Platelets (x10^9 /L)	251 (24-663)	C4 (g/L)	0.28 (0.13-7.39)	
Total proteins (g/L)	65 (45-89)	IgG (g/L)	6.55 (0.98-33.8)	
Albumin (g/L)	37 (21-300)	IgA (g/L)	0.63 (0.05-4.81)	
CRP (mg/L)	18.75 (1.6-1600)	IgM (g/L)	0.63 (0.06-6.47)	

a Includes: acute myeloid leukemia, acute lymphoblastic leukemia, myelodysplastic syndrome, chronic myeloid leukemia and myelofibrosis.

b Includes: Hodgkin's and non-Hodgkin's lymphoma, chronic lymphocytic leukemia and multiple myeloma.

c Includes: sarcomas and immunodeficiencies, aplastic anemia, paroxysmal nocturnal hemoglobinuria and other diagnoses.

d Clinician’s impression of disease activity was recorded for 120 patients.

e Patients either did not receive any immunosuppressive therapy or did not meet any of criteria or alter therapy due to toxicity.

ROC curves depicting previously described variables were used to describe specificity and sensitivity of glycans’ discriminatory potential. ROC curves for glycan measurements model were compared to ROC curves built on laboratory parameters model (including markers of inflammation), and finally to the ones combining both of the mentioned. An area below the ROC curve greater than 0.9 indicates an excellent model, while an area of 0.8-0.9 is considered very good.

## Results

### Patient Characteristics

Out of initially 262 blood plasma samples of cGvHD patients received, 20 samples were excluded because of the contamination (2) or unsatisfactory sample quality and/or low IgG concentration (17). Another 29 samples were excluded because of IVIg treatment within 3 months of sample collection.

Therefore, IgG glycome composition was analyzed in 213 cGvHD patients [56.8% male; median age 45 years (range 5-71)]. Majority received myeloablative conditioning (56.8%), had related donors (60.1%), peripheral blood hematopoietic stem cells source (77.9%), and history of a previous acute GvHD (66.2%). Most subjects were transplanted because of leukemia (64.8%) or lymphoproliferative disease (28.2%). These characteristics and variables describing the nature of their cGvHD are given in **Table 1**. Laboratory markers of inflammation (15) and other laboratory parameters listed in the literature as relevant to cGvHD are also presented in **Table 1**, while the rest of them are given in the supplemental material (**Table S1**).

Glycan measurements were tested against all of the patients’ characteristics and information on the onset and course of cGvHD listed in **Table 1** and did not yield any significant results.

### UHPLC Glycan Analysis

#### NIH cGvHD Organ Scores

Statistically significant values were observed when comparing the glycans of cGvHD patients with no joint/fascia affected by cGvHD versus those with NIH grade 1 (mild), NIH grade 2 (moderate) or NIH grade 3 (severe) cGvHD of joint/fascia, irrelevant of other organ involvement. The results of the direct measurements as well as the derivative properties show a significant decrease in the proportion of galactosylated structures with higher NIH score.

**Figure 1:**
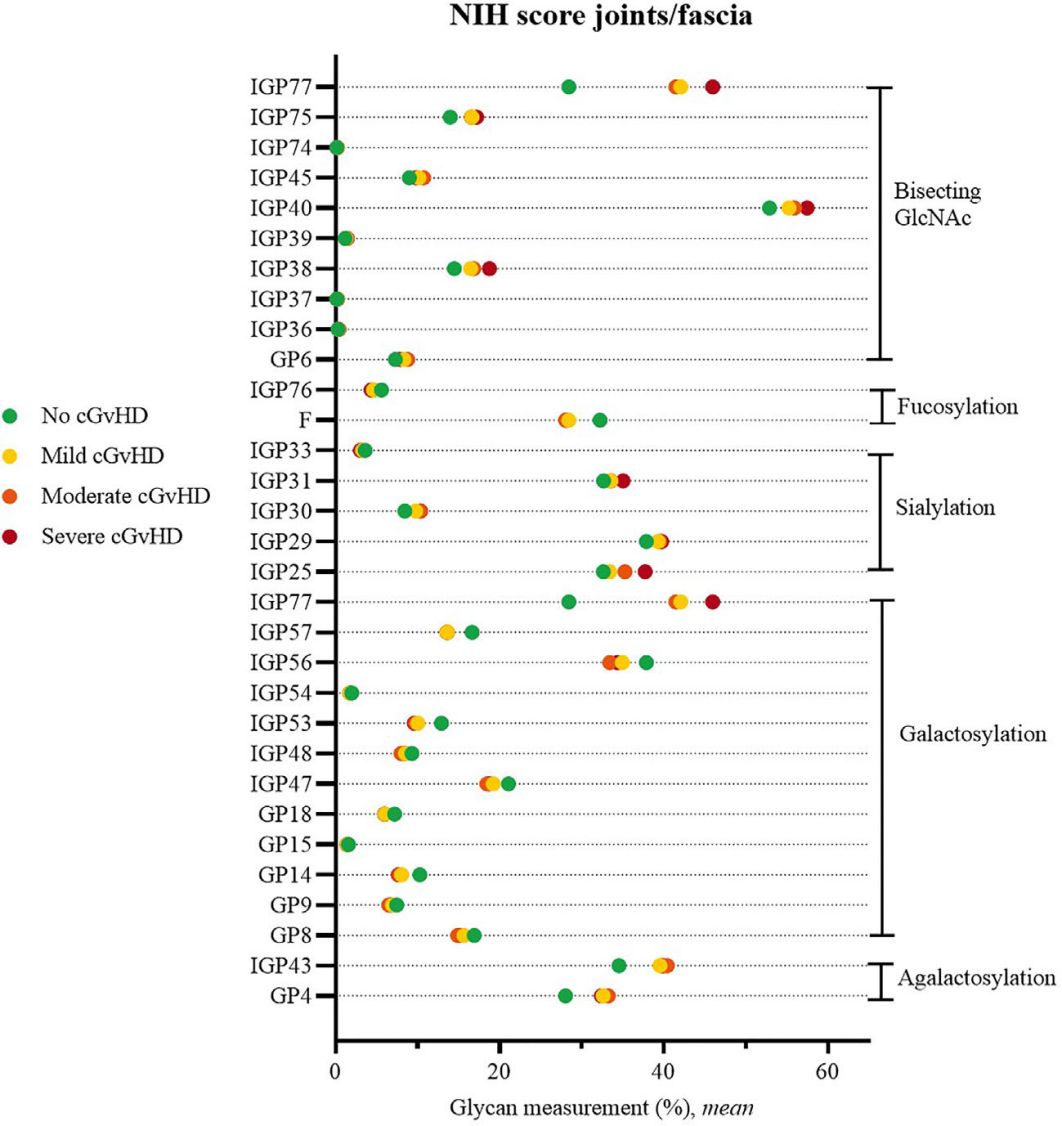
Significant differences in glycan measurements in patients with no joint/fascia cGvHD and among various degrees of joint/fascia NIH cGvHD scores.

Change was often observed in two or even all three degrees of severity of joint/fascia involvement for the following galactosylated glycan structures GP8, GP14, GP15, GP18, IGP47, IGP48, IGP53, IGP54, IGP56, IGP57. The analysis indicated an increase in the proportion of agalactosylated structures (GP4, IGP43). An increase in the proportion of sialylated structures was also observed (IGP25, IGP29, IGP30, IGP31, IGP33), as well as a decrease in the proportion of fucosylated structures (F, IGP76). Statistically significant, higher proportion of directly measured structures with branched GlcNAc and derived parameters suggesting an increase in its proportion were also characteristic for the group of patients with joint/fascia cGvHD (GP6, IGP36, IGP37, IGP38, IGP39, IGP40, IGP45, IGP74, IGP75, IGP77) (**Figure 1**).

**Figure 2:**
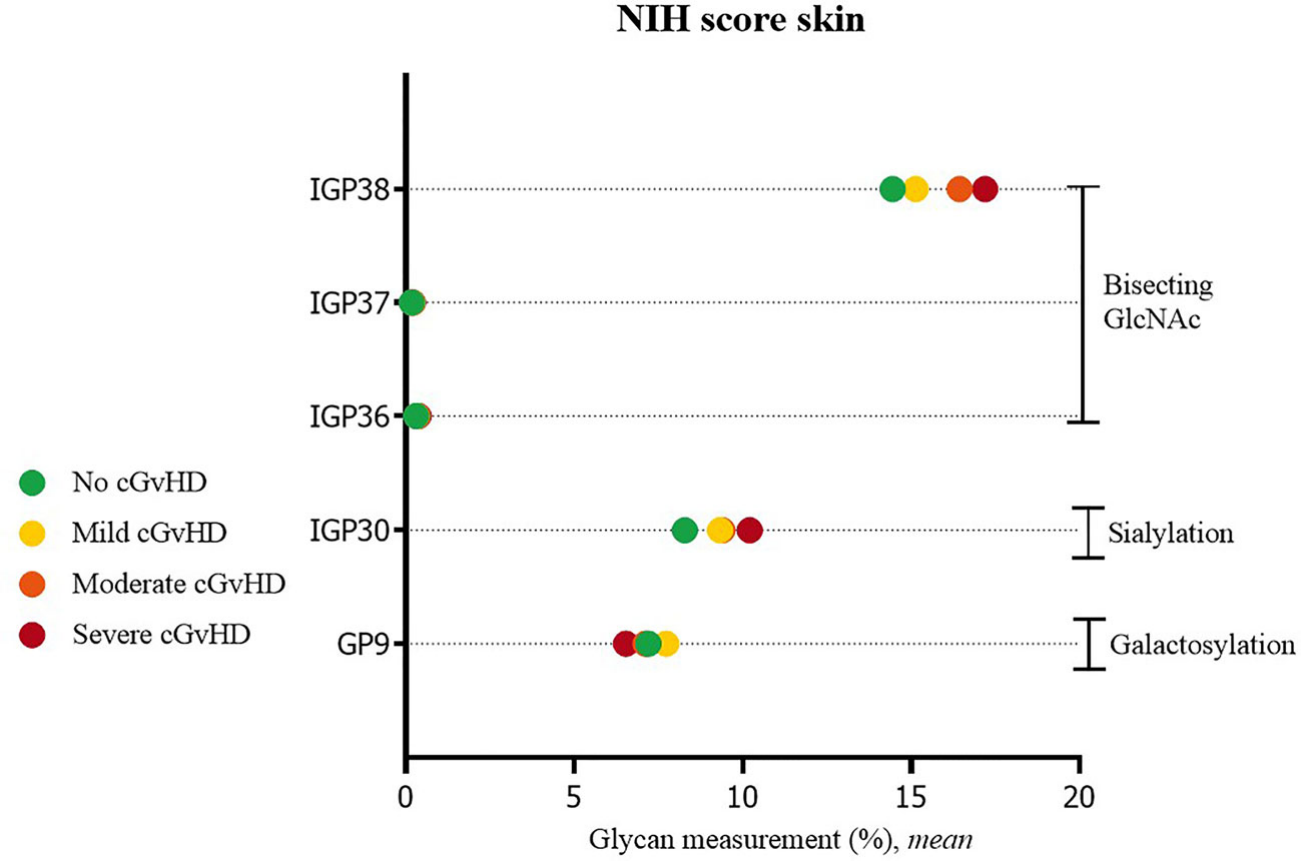
Significant differences in glycan measurements in patients with no skin cGvHD and among various degrees of skin NIH cGvHD severity.

Significant differences were also observed in cGvHD patients without skin cGvHD compared to patients with mild or severe skin NIH cGvHD scores. In patients with skin cGvHD, the proportion of structures with bisecting GlcNAc was statistically significantly increased (IGP36; IGP37, IGP38). A significantly higher proportion was also observed for IGP30 (disialylated of fucosylated digalactosylated structures without bisecting GlcNAc), whereas GP9, the monogalactosylated structure, showed the opposite trend (**Figure 2**). The complete list of all significant results of the UHPLC analysis is given in **Supplementary Table S2**. None of the other patients’ NIH cGvHD scores showed any significant differences in glycan measurements (data not shown).

#### CGvHD Activity

**Figure 3:**
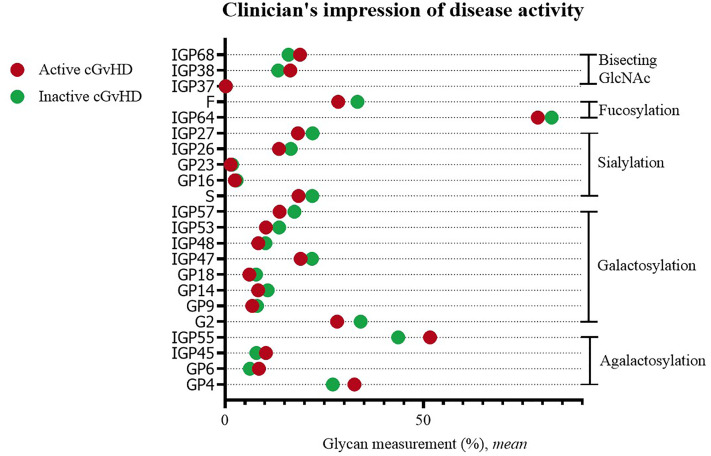
Significant differences in glycan measurements in patients with active and inactive cGvHD (by clinician’s impression).

Statistically significant differences were observed when comparing IgG glycans of patients with active versus inactive disease (by clinician’s impression). Inactive disease was characterized by a decreased proportion of agalactosylated structures (GP4, GP6, IGP45, IGP55), as well as an increased proportion of galactosylated structures (monogalactosylated: GP9, GP18, IGP47, IGP48; digalactosylated: G2, GP14, IGP53, IGP57). An increased proportion of sialylated (S, GP16, GP23, IGP26, IGP27) and fucosylated structures (F, IGP64), and a decreased proportion of structures with bisecting GlcNAc (IGP37, IGP38, IGP68) were characteristic for inactive disease (**Figure 3**).

**Figure 4:**
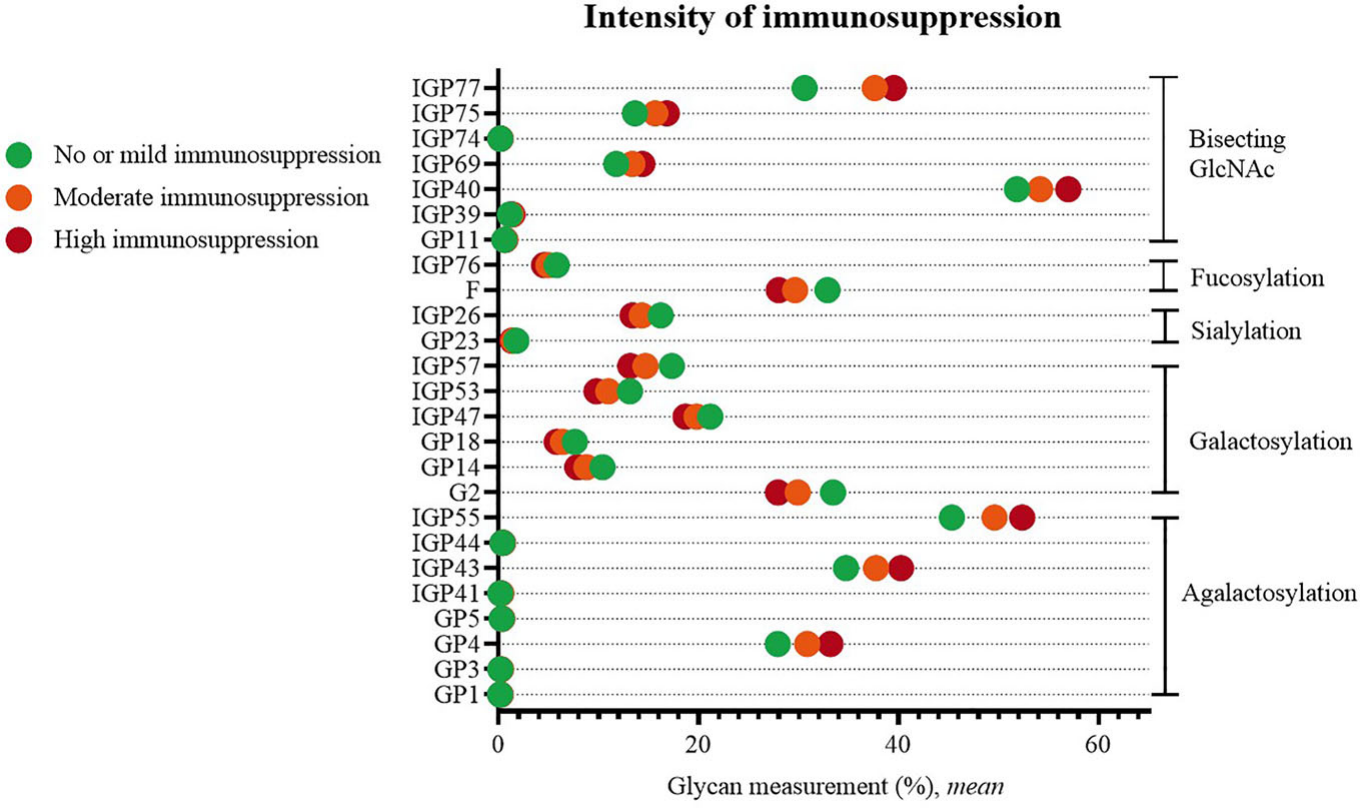
Significant differences in glycan measurements in patients of different degrees of systemic immunosuppression.

Statistically significant changes were visible when comparing patients with no or mild versus patients with moderate or high systemic immunosuppression. Higher immunosuppression was characterized by an increased proportion of agalactosylated structures (GP1, GP3, GP4, GP5, IGP41, IGP43, IGP44, IGP55), and structures with bisecting GlcNAc (GP11, IGP39, IGP40, IGP69, IGP74, IGP75, IGP77), as well by a decreased proportion of galactosylated (G2, GP14, GP18, IGP47, IGP53, IGP57), sialylated (GP23, IGP26) and fucosylated structures (F, IGP76) (**Figure 4**).

No statistical differences were detected when comparing cGvHD activity by therapeutic intent at the time of evaluation (data not shown).

The complete list of significant results of the UHPLC analysis is given in **Supplementary Table S2**.

### Logistic Regression Analysis and ROC Curves

#### NIH cGvHD Organ Scores

Logistic regression models were built for: A) glycan measurements; B) laboratory parameters (including markers of inflammation), and C) glycan measurements and laboratory parameters combined. These models created for involvement of individual NIH cGvHD organ scores and cGvHD activity were evaluated and compared by using ROC curves which describe specificity and sensitivity of glycans’ discriminatory potential.

The ROC curves of glycan measurements (by the means of PCA) show the following values: NIH cGvHD skin (0.6922), NIH cGvHD mouth (0.6121), NIH cGvHD eyes (0.5732), NIH cGvHD gastrointestinal system (0.6238), NIH cGvHD liver (0.6626), NIH cGvHD lungs (0.6176), NIH cGvHD joints/fascia (0.7263) and NIH cGvHD genital in women (0.5999). Upgrading the model built for laboratory parameters (PCA) by combining glycan measurements shows an increase in the area under the ROC curve of all individual NIH cGvHD organ systems affected by cGvHD: skin (0.7968 → 0.8271), mouth (0.7882 → 0.7932), eyes (0.6779 → 0.7181), gastrointestinal system (0.7149 → 0.7872), liver (0.9200 → 0.9344), lungs (0.7419 → 0.7654), joints/fascia 0.8056 → 0.8668) and the genital system in women (0.8355 → 0.8636).

#### CGvHD Activity

**Figure 5:**
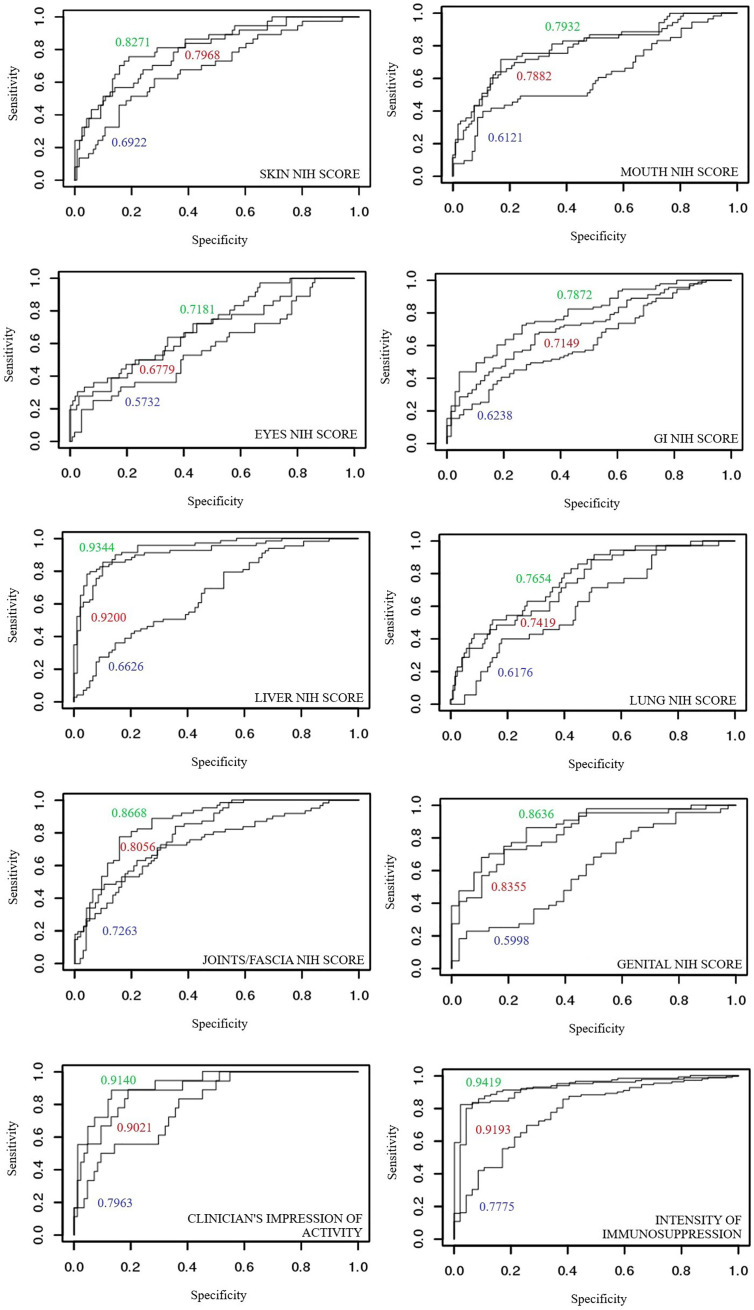
Comparison of areas under ROC curves for discriminant models of individual organ systems with and without cGvHD: (blue) glycans measurements, (red) laboratory parameters and (green) model combining laboratory parameters and glycan measurements.

The ROC curves of glycan measurements for cGvHD activity show the following results: 0.7953 for the clinician’s impression of disease activity and 0.7775 for intensity of immunosuppression. Upgrading the model built by using the main components of laboratory parameters into a model that includes glycan measurements shows an increase in the area below the ROC curve for cGvHD activity parameters: clinician’s impression of disease activity (0.9021 → 0.9140) and intensity of immunosuppression (0.9192 → 0.9418). ROC curves are shown in **Figure 5**.

## Discussion

The results of this study for the first time confirm that N-glycosylation of IgG from plasma of well-defined and evaluated cGvHD patients’ cohort is associated with clinical manifestations of cGvHD.

UHPLC glycan analysis of blood plasma samples showed a significant association of individual glycans and changes in their composition with joints/fascia and skin cGvHD, as well as with clinician’s impression of cGvHD activity and intensity of immunosuppression of cGvHD patients.

### CGvHD of Joints/Fascia and Skin

A significant decrease in the proportion of directly measured glycans were observed in patients with cGvHD of joints/fascia. The decrease in the proportion of galactosylated glycans was also visible through by a decrease in the derivative parameters representing the proportion or frequency of glycan structures with one or two galactoses. Particularly interesting glycan could be FA2G2 (GP14) which significant decrease can be observed through all three degrees of joint/fascia cGvHD severity. A decrease in IgG galactosylation has been repeatedly associated with rheumatoid arthritis (17–19), while recent studies even indicate that a change in IgG glycan composition can be detected several years before onset and indicates an increased risk of developing the disease (20). The analysis also showed a significant increase in the proportion of GP4 (FA2), an agalactosylated structure. De Jong et al. (21) also describe reduced IgG1 galactosylation, and they consider it to be a biomarker of immune system activation. Additionally, agalactosylated glycans are associated with increased affinity of IgG for FcγRIII (activating receptor) and mannose binding lectin resulting in complement activation (22, 23). The association of the C3 complement component with joints/fascia cGvHD was described in an earlier study (16), and recently confirmed in another population (24). Elevated CRP, a marker of inflammation associated with cGvHD activity (16) was also recently associated to reduced galactosylation (25).

Although IgG sialylation dynamics usually follows galactosylation since the existence of galactose is a prerequisite for the binding of terminal sialic acid, an increase in the proportion of structures with sialic acid was observed in our study. The increase was not determined by measuring direct properties but was evident from derivative properties that describe the sialylation of galactosylated fucosylated structures with or without bisecting GlcNAc.

A reduced proportion of IgG Fc galactosylation was recently described in a longitudinal pediatric study where the authors observed lower levels of galactosylation in transplant patients (both before and after HSCT) versus HSC donors and healthy controls (26). The change was particularly pronounced in the group of patients transplanted for malignant diagnosis (leukemia). Patients with acute or chronic GvHD, IVIg-dependent patients, as well as those on immunosuppressive therapy 7 months after alloHSCT were excluded from this study. The same study described an increased sialylation per galactose unit which was attributed to the increased extrinsic sialyltransferase activity of ST6Gal1 due to radiation or inflammation, and increased availability of CMP sialic acid released upon activation of circulating platelets during the inflammatory process. As most cGvHD patients in this study were transplanted for malignant diagnosis (leukemia), the observed reduced galactosylation and/or elevated sialylation cannot be exclusively attributed to cGvHD. Other observed changes in glycan composition may be due to cGvHD or immunosuppressive therapy.

Unusual dynamics of the sialic acid content could also be explained by its origin from a Fab rather than an Fc fragment of IgG. Studies have shown that in autoimmune (rheumatoid arthritis, Sjögren’s syndrome) but also in malignant diseases (myeloma, Burkitt’s lymphoma, follicular and diffuse B-cell lymphoma), the number of potential glycan binding sites on the Fab fragment increases (27). Authors Holland et al. (28) in their study of ANCA-vasculitis describe hypogalactosylation restricted to the Fc fragment of IgG while the Fab fragment is galactosylated and sialylated. Compared to Fc glycans, Fab glycans are more exposed to glycosidase and glycosyl-transferase activity due to its position, and more frequently tends to bind bisecting GlcNAc, galactose and sialic acid to its less fucosylated heptasaccharide bianntenary core.

An increase in the total proportion of bisecting GlcNAc is associated with an increased affinity for FcγRIIIa and consequently increased antibody-dependent cellular cytotoxicity (ADCC) (29). Simultaneously, a reduced proportion of IgG core fucose was measured, which is thought to have a reciprocal effect from the presence of bisecting GlcNAc (decreasing the affinity for the same receptor). Both measurements speak in favor of increased ADCC. The same was previously observed in systemic lupus erythema (30), a chronic multisystemic disease resembling cGvHD. The study also noted a significant decrease in the aforementioned GP14 (FA2G2), which is therefore considered a good candidate for the predictive biomarker of the disease. GP14 is a glycan that has also been repeatedly linked to ulcerative colitis where it correlates to disease clinical course and its activity (31), and could play a valuable role in disease discrimination. In the same study, GP9 (FA2G1) is attributed the highest predictive value in the analyzed populations, while the authors consider the decrease in galactosylation of IgG and the consequent alteration of the antibodies inflammatory potential as one of the molecular mechanisms involved in systemic lupus erythematosus pathophysiology and/or autoimmunity.

In our study, similar to changes observed in joints/fascia cGvHD, the trend of decreasing galactosylation is also observed in skin cGvHD, while there is an increase in the proportion of sialylation and bisecting GlcNAc. Skin cGvHD is known to coincide with cGvHD of joints/fascia (32), and it is known that the sclerotic form of skin cGvHD significantly reduces joint movement as well as the grip force (33). The association of skin and joints/fascia cGvHD was also confirmed in a recent study in the Croatian cGvHD population (23). It is therefore not unexpected that changes in the IgG glycan profile of skin cGvHD follow the pattern of joints/fascial cGvHD-related changes.

### CGvHD Activity

Regarding cGvHD activity, analysis of the samples indicated a lower proportion of agalactosylated glycan structures, as well as a higher proportion of galactosylated structures in plasma samples of patients whose cGvHD was considered non-active. The same samples are characterized by more sialylated and fucosylated structures and a smaller proportion of structures with bisecting GlcNAc compared to the samples of subjects with active cGvHD. Described changes are a possible consequence of the disproportionate number of active cases (101, median age 46.5, range 5-71) versus non-active cases of cGvHD (19, median age 25, range 7-66), as well as the age difference in the two groups. Older age was previously linked with the decrease in the concentration of galactosylated structures (14).

Another indicator of cGvHD activity showed consistent results with the clinical impression of disease activity. In general, statistically significantly higher proportions of agalactosylated structures, reduced proportions of galactosylated and sialylated structures were identified in patients with moderate or high intensity systemic immunosuppression compared to cGvHD patients with mild or no immunosuppression. The increased proportion of glycan structures with bisecting GlcNAc and decreased proportions of fucosylated structures also coincide with the clinical impression of disease activity. Particularly interesting were the structures GP1 (FA1) and GP3(A2B), which were found to be significantly elevated in patients with moderate and high levels of systemic immunosuppression, thus indicating a potentially greater sensitivity to the therapy used and, indirectly, to increased cGvHD activity.

### Limitations of the Study

Limitations of this study are relatively small number of patients, cross-sectional design of the study, long time after HSCT and long time after cGvHD diagnosis to plasma IgG glycan analysis, including majority of patients with severe and active disease with several previous lines of systemic therapy and intensive current immunosuppression. However, it is a well defined and thoroughly evaluated cohort of cGvHD patients, with many clinical and laboratory details taking into consideration regarding glycan analyses.

### Glycans as a Potential Biomarker for cGvHD

The specificity and sensitivity of glycan measurements as a potential diagnostic test for cGvHD organ involvement as well as disease activity were tested using ROC curves. The ROC curves of glycan measurements were compared with models built using laboratory markers of inflammation, as well as a model that combines both. The analysis found that glycan measurements, although insufficiently specific and sensitive on their own, successfully upgrade models constructed using laboratory markers of inflammation, contributing to a greater predictive ability to discriminate healthy organ systems from those affected by cGvHD. This applies to all organ systems affected with cGvHD (skin, mouth, eyes, digestive tract, liver, lungs, joints/fascia, genital tract in women) as well for scores describing disease activity. In most cases, very good models based on laboratory markers of inflammation, are upgraded to excellent models of high specificity and sensitivity when combined with glycan measurements.

The results of this study for the first time confirm significant associations of IgG glycan structures with cGvHD manifestations, which may contribute to understanding the biology of the disease and lead to the development of a potential biomarker of cGvHD. Further studies are required, preferentially in longitudinal post-transplant cohorts, in order to validate these associations, determine the effect of post-transplant immunosuppression and treatment, and evaluate the potential practical clinical application in the diagnosis and treatment of cGvHD.

## Data Availability Statement

The raw data supporting the conclusions of this article will be made available by the authors, without undue reservation.

## Ethics Statement

The studies involving human participants were reviewed and approved by Center for Cancer Research, National Institutes of Health Ethics Committee. Written informed consent to participate in this study was provided by the participants’ legal guardian/next of kin.

## Author Contributions

EP was the principal researcher and executor of the experiment and paper - this paper is part of her dissertation. DP and SP (also the principal investigator of the clinical study) mentored this research and were clinical researchers of the study. GL, together with previous authors, contributed to the research idea and concept. LD, FP, SM, RV, and DN were part of the clinical research team, also in charge of cGvHD evaluation. IU and MM conducted the statistical analysis. MP-B contributed and supervised the experimental glycan analysis. JR conducted the initial analysis and storage of cGvHD plasma samples. All authors contributed to the article and approved the submitted version.

## Funding

This work is supported by the Unity Through Knowledge Fund project entitled “Clinical and Biological Factors Determining Severity and Activity of Chronic Graft-Versus-Host Disease after Allogeneic Hematopoietic Stem Cell Transplantation”, and also, in part, by the Croatian Science Foundation project IP-2016-06-8046 entitled “New biomarkers for chronic Graft-versus-Host disease”. This work is supported by the Center for Cancer Research, Intramural Research Program of the National Cancer Institute, National Institutes of Health, USA. The opinions expressed here are those of the authors and do not represent the official position of the National Institutes of Health or the US Government.

## Conflict of Interest

Author EP is currently employed by the company Fidelta Ltd. Authors MP-B, IU and GL were employed by the company Genos Ltd.

The remaining authors declare that the research was conducted in the absence of any commercial or financial relationships that could be construed as a potential conflict of interest.
