# Supplementary material for: Significant Associations of IgG Glycan Structures With Chronic Graft-Versus-Host Disease Manifestations: Results of the Cross-Sectional NIH Cohort Study
Source: Front Immunol. 2021 Jul 14;12:633214. doi: 10.3389/fimmu.2021.633214 (PMC8317462; doi:10.3389/fimmu.2021.633214)
Supplement: Supplementary file 1 [file DataSheet_1.doc]

Supplement

**Table S1.** Complete list of laboratory parameters of cGvHD patients.

| **Laboratory parameters** | **Median (range)** | **Laboratory parameters** | **Median (range)** |
| --- | --- | --- | --- |
| Erythrocyte sedimentation (mm/h) | 16 (1-113) | Aspartate aminotransferase (U/L) | 31 (8-256) |
| Erythrocytes (x10^12/L) | 4.10 (0.29-62.80) | Alanine aminotransferase (U/L) | 40 (12-324) |
| Hemoglobin (g/L) | 126 (84-180) | Gamma-glutamyltransferase (U/L) | 81 (7-3077) |
| Hematocrit (L/L) | 0.39 (0.22-0.54) | Creatine kinase (U/L) | 56 (12-387) |
| Reticulocytes (x10^9 /L) | 62.70 (1.74-443) | Lactate dehydrogenase (U/L) | 212 (105-1078) |
| Leukocytes (x10^9 /L) | 7.55 (2.28-31.30) | Calcium (mmol/L) | 2.29 (1.54-2.84) |
| Lymphocytes (x10^9 /L) | 1.34 (0.15-9.79) | CRP (mg/L) | 18.75 (1.6-1600) |
| Neutrophils (x10^9 /L) | 4.85 (0.86-333.09) | Ferritin (μg/L) | 409 (8-5604) |
| Eosinophils (x10^9 /L) | 0.08 (0-91.07) | Total cholesterol (mmol/L) | 5.28 (0.03-261.71) |
| Platelets (x10^9 /L) | 251 (24-663) | Triglycerides (mmol/L) | 2.15 (0.33-9.46) |
| T lymphocytes (number of cells) | 800 (56-6775) | HDL (mmol/L) | 1.47 (0.004-34.70) |
| T helper lymphocytes (number of cells) | 377.50 (14-2420) | LDL (mmol/L) | 3.03 (0.51-33.80) |
| Cytotoxic T lymphocytes (number of cells) | 330 (29-4833) | Beta 2 microglobulin (mg/L) | 2.10 (0.9-70.74) |
| B lymphocytes (number of cells) | 114 (0-6307) | Total proteins (g/L) | 65 (45-89) |
| NK cells (number of cells) | 180.5 (14-996) | Albumin (g/L) | 37 (21-300) |
| PV | 1.30 (0.36-6.36) | IgG (g/L) | 6.55 (0.98-33.8) |
| APTV (s) | 29.60 (13.1-190.34) | IgA (g/L) | 0.63 (0.05-4.81) |
| Glucose (mmol/L) | 5.99 (0.07-83.11) | IgM (g/L) | 0.63 (0.06-6.47) |
| Bilirubin (µmol/L) | 8.55 (1.71-285.50) | Rheumatoid factor (nmol/L) | 10 (10-288) |
| Urea (mmol/L) | 5.71 (0.14-297.40) | C3 (g/L) | 1.35 (0.64-2.22) |
| Urate (µmol/L) | 255.76 (2.69-594.80) | C4 (g/L) | 0.28 (0.13-7.39) |
| Creatinine (µmol/L) | 72.50 (1.71-583.57) | TSH (mIJ/L) | 1.36 (0.02-35.6) |
| Alkaline phosphatase (U/L) | 98 (27-1004) | PTH (pmol/L) | 4.28 (0.50-47.04) |

**Table S2.** Complete list of significant results (in bold) of the UHPLC analysis for joints/fascia and skin severity scores, as well as clinician’s impression of cGvHD activity and intensity of immunosuppression.

| **Severity of chronic GvHD** | | | | | | | | |
| --- | --- | --- | --- | --- | --- | --- | --- | --- |
| **NIH score of joints/fascia:**  **(0) None; (1) Mild; (2) Moderate; (3) Severe** | | | | | | | | |
| **Glycan structures** | | **Comparison of NIH score 0-1** | | **Comparison of NIH score 0-2** | | **Comparison of NIH score 0-3** | | **Increase ↑/ decrease ↓** |
|  |  | **p-value** | | | | | |  |
| **GP4** | | **0.0399** | | **0.0182** | | 0.0888 | | **↑** |
| **GP6** | | 0.2125 | | **0.0420** | | 0.4417 | | **↑** |
| **GP8** | | 0.0934 | | **0.0045** | | 0.0994 | | **↓** |
| **GP9** | | 0.4725 | | **0.0182** | | 0.1385 | | **↓** |
| **GP14** | | **0.0228** | | **0.0028** | | **0.0028** | | **↓** |
| **GP15** | | 0.4005 | | **0.0386** | | 0.4725 | | **↓** |
| **GP18** | | **0.0359** | | **0.0252** | | 0.0545 | | **↓** |
| **IGP25** | | 0.9330 | | 0.5347 | | **0.0182** | | **↑** |
| **IGP29** | | 0.2365 | | **0.0182** | | 0.2634 | | **↑** |
| **IGP30** | | 0.0994 | | **0.0252** | | **0.0253** | | **↑** |
| **IGP31** | | 0.4416 | | 0.2250 | | **0.0339** | | **↑** |
| **IGP33** | | 0.1270 | | 0.0567 | | **0.0355** | | **↓** |
| **IGP36** | | 0.0810 | | **0.0238** | | **0.0045** | | **↑** |
| **IGP37** | | 0.1300 | | **0.0253** | | **0.0028** | | **↑** |
| **IGP38** | | 0.1300 | | **0.0253** | | **0.0028** | | **↑** |
| **IGP39** | | 0.3991 | | **0.0435** | | 0.0636 | | **↑** |
| **IGP40** | | 0.3991 | | **0.0435** | | 0.0636 | | **↑** |
| **IGP43** | | **0.0359** | | **0.0057** | | **0.0434** | | **↑** |
| **IGP45** | | 0.2548 | | **0.0433** | | 0.5352 | | **↑** |
| **IGP47** | | 0.0639 | | **0.0045** | | 0.0801 | | **↓** |
| **IGP48** | | 0.3371 | | **0.0086** | | 0.1645 | | **↓** |
| **IGP53** | | **0.0230** | | **0.0045** | | **0.0182** | | **↓** |
| **IGP54** | | 0.3557 | | **0.0457** | | 0.5206 | | **↓** |
| **IGP55** | | **0.0419** | | **0.0057** | | **0.0359** | | **↑** |
| **IGP56** | | 0.0702 | | **0.0028** | | **0.0435** | | **↓** |
| **IGP57** | | **0.0315** | | **0.0237** | | 0.0548 | | **↓** |
| **IGP74** | | **0.0440** | | **0.0352** | | **0.0352** | | **↑** |
| **IGP75** | | **0.0440** | | **0.0352** | | **0.0352** | | **↑** |
| **IGP76** | | **0.0434** | | **0.0228** | | **0.0333** | | **↓** |
| **IGP77** | | 0.0586 | | **0.0228** | | **0.0404** | | **↑** |
| **F** | | **0.0435** | | **0.0182** | | **0.0420** | | ↓ |
|  | | | | | | | | |
| **NIH score of skin:**  **(0) None; (1) Mild; (2) Moderate; (3) Severe** | | | | | | | | |
| **Glycan structure** | **NIH score** | | **NIH score** | | **Increase ↑/ decrease ↓** | | **p-value** | |
| **IGP36** | 0 | | 3 | | ↑ | | **0.0474** | |
| **IGP37** | 0 | | 3 | | ↑ | | **0.0474** | |
| **IGP38** | 0 | | 3 | | ↑ | | **0.0474** | |
| **IGP30** | 0 | | 3 | | ↑ | | **0.0474** | |
| **GP9** | 1 | | 3 | | ↓ | | **0.0474** | |

| **Activity of chronic GvHD** | | | | |
| --- | --- | --- | --- | --- |
| **Clinician's impression of activity**  **(1) Active disease; (2) Inactive disease** | | | | |
| **Glycan structure** | **Clinician's impression of activity** | **Clinician's impression of activity** | **Increase ↑/ decrease ↓** | **p-value** |
| **GP23** | 1 | 2 | ↑ | **0.0192** |
| **G2** | 1 | 2 | ↑ | **0.0192** |
| **IGP48** | 1 | 2 | ↑ | **0.0192** |
| **GP6** | 1 | 2 | ↓ | **0.0192** |
| **S** | 1 | 2 | ↑ | **0.0192** |
| **IGP26** | 1 | 2 | ↑ | **0.0192** |
| **GP16** | 1 | 2 | ↑ | **0.0192** |
| **GP18** | 1 | 2 | ↑ | **0.0192** |
| **IGP45** | 1 | 2 | ↓ | **0.0217** |
| **IGP55** | 1 | 2 | ↓ | **0.0218** |
| **IGP27** | 1 | 2 | ↑ | **0.0218** |
| **IGP37** | 1 | 2 | ↓ | **0.0218** |
| **IGP38** | 1 | 2 | ↓ | **0.0218** |
| **IGP56** | 1 | 2 | ↑ | **0.0218** |
| **IGP53** | 1 | 2 | ↑ | **0.0218** |
| **GP9** | 1 | 2 | ↑ | **0.0222** |
| **IGP57** | 1 | 2 | ↑ | **0.0222** |
| **IGP47** | 1 | 2 | ↑ | **0.0222** |
| **F** | 1 | 2 | ↑ | **0.0247** |
| **GP14** | 1 | 2 | ↑ | **0.0304** |
| **GP4** | 1 | 2 | ↓ | **0.0433** |
| **IGP64** | 1 | 2 | ↑ | **0.0477** |
| **IGP68** | 1 | 2 | ↓ | **0.0484** |
|  |  |  |  |  |
| **Intensity of immunosuppression**  **(1) None; (2) Mild; (3) Moderate; (4) High** | | | | |
| **Glycan structure** | **Intensity of immunosuppression** | **Intensity of immunosuppression** | **Increase ↑/ decrease ↓** | **p-value** |
| **GP3** | 1 | 4 | ↑ | **0.0012** |
| **GP1** | 1 | 3 | ↑ | **0.0012** |
| **IGP41** | 1 | 3 | ↑ | **0.0012** |
| **GP1** | 1 | 4 | ↑ | **0.0012** |
| **IGP41** | 1 | 4 | ↑ | **0.0012** |
| **GP18** | 1 | 4 | ↓ | **0.0012** |
| **GP23** | 1 | 4 | ↓ | **0.0033** |
| **IGP57** | 1 | 4 | ↓ | **0.0039** |
| **IGP26** | 1 | 4 | ↓ | **0.0042** |
| **GP3** | 1 | 3 | ↑ | **0.0042** |
| **IGP53** | 1 | 4 | ↓ | **0.0042** |
| **IGP69** | 1 | 4 | ↑ | **0.0042** |
| **GP14** | 1 | 4 | ↓ | **0.0045** |
| **G2** | 1 | 4 | ↓ | **0.0061** |
| **F** | 1 | 4 | ↓ | **0.0063** |
| **GP5** | 1 | 4 | ↑ | **0.0077** |
| **IGP39** | 1 | 4 | ↑ | **0.0089** |
| **IGP40** | 1 | 4 | ↑ | **0.0089** |
| **IGP55** | 1 | 4 | ↑ | **0.0151** |
| **GP5** | 1 | 3 | ↑ | **0.0151** |
| **IGP74** | 1 | 4 | ↑ | **0.0151** |
| **IGP75** | 1 | 4 | ↑ | **0.0151** |
| **IGP76** | 1 | 4 | ↓ | **0.0174** |
| **IGP44** | 1 | 4 | ↑ | **0.0179** |
| **IGP43** | 1 | 4 | ↑ | **0.0251** |
| **GP4** | 1 | 4 | ↑ | **0.0259** |
| **IGP77** | 1 | 4 | ↑ | **0.0268** |
| **IGP47** | 1 | 4 | ↓ | **0.0268** |
| **IGP44** | 1 | 3 | ↑ | **0.0359** |
| **GP11** | 1 | 4 | ↑ | **0.0359** |
| **GP23** | 1 | 3 | ↓ | **0.0359** |
| **GP18** | 1 | 3 | ↓ | **0.0362** |
